# Association Between Implementation of the Severe Sepsis and Septic Shock Early Management Bundle Performance Measure and Outcomes in Patients With Suspected Sepsis in US Hospitals

**DOI:** 10.1001/jamanetworkopen.2021.38596

**Published:** 2021-12-20

**Authors:** Chanu Rhee, Tingting Yu, Rui Wang, Sameer S. Kadri, David Fram, Huai-Chun Chen, Michael Klompas

**Affiliations:** 1 Department of Population Medicine, Harvard Medical School, Harvard Pilgrim Health Care Institute, Boston, Massachusetts; 2 Division of Infectious Diseases, Department of Medicine, Brigham and Women’s Hospital, Boston, Massachusetts; 3 Critical Care Medicine Department, National Institutes of Health Clinical Center, Bethesda, Maryland; 4 Commonwealth Informatics, Waltham, Massachusetts

## Abstract

**Importance:**

In October 2015, the Centers for Medicare & Medicaid Services began requiring US hospitals to report adherence to the Severe Sepsis and Septic Shock Early Management Bundle (SEP-1).

**Objective:**

To evaluate the association of SEP-1 implementation with sepsis treatment patterns and outcomes in diverse hospitals.

**Design, Setting, and Participants:**

This retrospective cohort study with interrupted time-series analysis and logistic regression models was conducted among adults admitted to 114 hospitals from October 2013 to December 2017 with suspected sepsis (blood culture orders, ≥2 systemic inflammatory response syndrome criteria, and acute organ dysfunction) within 24 hours of hospital arrival. Data analysis was conducted from September 2020 to September 2021.

**Exposures:**

SEP-1 implementation in the fourth quarter (Q4) of 2015.

**Main Outcomes and Measures:**

The primary outcome was quarterly rates of risk-adjusted short-term mortality (in-hospital death or discharge to hospice). Secondary outcomes included lactate testing and administration of anti–methicillin-resistant *Staphylococcus aureus* (MRSA) or antipseudomonal β-lactam antibiotics within 24 hours of hospital arrival. Generalized estimating equations with robust sandwich variances were used to fit logistic regression models to assess for changes in level or trends in these outcomes, adjusting for baseline characteristics and severity of illness.

**Results:**

The cohort included 117 510 patients (median [IQR] age, 67 years [55-78] years; 60 530 [51.5%] men and 56 980 [48.5%] women) with suspected sepsis. Lactate testing rates increased from 55.1% (95% CI, 53.9%-56.2%) in Q4 of 2013 to 76.7% (95% CI, 75.4%-78.0%) in Q4 of 2017, with a significant level change following SEP-1 implementation (odds ratio [OR], 1.34; 95% CI, 1.04-1.74). There were increases in use of anti-MRSA antibiotics (19.8% [95% CI, 18.9%-20.7%] in Q4 of 2013 to 26.3% [95% CI, 24.9%-27.7%] in Q4 of 2017) and antipseudomonal antibiotics (27.7% [95% CI, 26.7%-28.8%] in Q4 of 2013 to 40.5% [95% CI, 38.9%-42.0%] in Q4 of 2017), but these trends preceded SEP-1 and did not change with SEP-1 implementation. Unadjusted short-term mortality rates were similar in the pre–SEP-1 period (Q4 of 2013 through Q3 of 2015) vs the post–SEP-1 period (Q1 of 2016 through Q4 of 2017) (20.3% [95% CI, 20.0%-20.6%] vs 20.4% [95% CI, 20.1%-20.7%]), and SEP-1 implementation was not associated with changes in level (OR, 0.94; 95% CI, 0.68-1.29) or trend (OR, 1.00; 95% CI, 0.97-1.04) for risk-adjusted short-term mortality rates.

**Conclusions and Relevance:**

In this cohort study, SEP-1 implementation was associated with an immediate increase in lactate testing rates, no change in already-increasing rates of broad-spectrum antibiotic use, and no change in short-term mortality rates for patients with suspected sepsis. Other approaches to decrease sepsis mortality may be warranted.

## Key Points

**Question:**

Was implementation of the CMS Severe Sepsis and Septic Shock Early Management Bundle (SEP-1) in October 2015 associated with improvements in sepsis-associated mortality?

**Findings:**

In this cohort study of 117 510 adult patients admitted to 114 US hospitals with clinical evidence of suspected sepsis between October 2013 and December 2017, SEP-1 implementation was associated with an immediate increase in lactate testing rates, no change in already-increasing rates of broad-spectrum antibiotic use, and no change in the combined outcome of in-hospital death or discharge to hospice.

**Meaning:**

These findings suggest SEP-1 was not associated with improved sepsis outcomes and that alternate approaches to preventing sepsis deaths in hospitals are needed.

## Teaser

This cohort study evaluates the association of implementation of the Severe Sepsis and Septic Shock Early Management Bundle (SEP-1) with sepsis treatment patterns and outcomes in US hospitals.

## Introduction

Sepsis is a leading cause of death, disability, and health care costs.^1,2^ This has triggered regulators and hospitals to invest heavily in improving sepsis recognition and care. Most notably, in October 2015, the Centers for Medicare & Medicaid Services (CMS) implemented the Severe Sepsis and Septic Shock Early Management Bundle (SEP-1) in requiring US hospitals to begin reporting adherence to a sepsis care bundle.^3^ The SEP-1 bundle requires clinicians to measure lactate, draw blood cultures, administer broad-spectrum antibiotics, and infuse 30 mL/kg or more of intravenous crystalloids (for patients with hypotension or lactate levels ≥4.0 mmol/L [to convert to milligrams per deciliter, divide by 0.111]) within 3 hours of sepsis time zero.^4^ Clinicians are further required to repeat lactate measurements if the initial lactate is greater than 2.0 mmol/L, initiate vasopressors, and document a repeat volume and perfusion assessment within 6 hours for patients with septic shock.

SEP-1 has catalyzed widespread sepsis quality improvement efforts, but concerns have been raised about its potential unintended consequences, including increasing inappropriate use of broad-spectrum antibiotics, overresuscitation with intravenous fluids, and diagnostic misdirection by overemphasizing sepsis to the exclusion of other serious diagnoses.^5,6,7,8,9,10,11,12,13^ Concerns have also been raised about the strength of evidence supporting the measure.^14^ SEP-1 is predominantly supported by observational studies reporting population-level decreases in sepsis-associated mortality after implementing sepsis bundles.^15,16,17,18,19,20^ However, bundle implementations are almost always accompanied by efforts to increase early sepsis recognition. This leads to the detection of milder cases of sepsis, thus making it difficult to determine whether improved mortality rates are owing to bundle implementations or because patients who are less severely ill are being included in sepsis case counts.^21,22^

Understanding whether and how SEP-1 has affected patients’ care and outcomes is critical for informing policy makers about the utility of the measure and how best to allocate future resources. However, to assess efficacy objectively, one must mitigate the ascertainment bias that occurs with sepsis quality improvement initiatives. One strategy to do so is to use stable clinical criteria to identify sepsis rather than diagnosis codes or sepsis registries, since these are subject to changes in diagnostic thresholds.^23,24^ We applied this strategy to detailed electronic clinical data from 114 US hospitals to assess the association of SEP-1 implementation with lactate testing, antibiotic use, and mortality in patients with suspected sepsis.

## Methods

This cohort study was approved by the Mass General Brigham institutional review board with a waiver of informed consent given the use of a deidentified commercial data source. Reporting followed the Strengthening the Reporting of Observational Studies in Epidemiology (STROBE) reporting guideline for cohort studies.

### Study Design and Data Source

This was a retrospective cohort study with interrupted time-series analysis and logistic regression models using the Cerner HealthFacts data set, a deidentified database with granular clinical data from geographically diverse US hospitals that use the Cerner electronic health record (EHR) system. Previous studies have demonstrated the utility of these data for sepsis analyses.^25,26^ We included all patients aged 18 years and older hospitalized between October 1, 2013, and December 31, 2017.

### Sepsis Clinical Criteria

Our primary study population was patients with suspected sepsis present on admission. We defined this group using clinical criteria mirroring SEP-1’s definition of severe sepsis to approximate the population directly targeted by the measure. Specifically, we defined suspected sepsis as all of the following within 24 hours of hospital arrival: (1) a blood culture order (regardless of result), (2) at least 2 systemic inflammatory response syndrome (SIRS) criteria (temperature >100.9 °F or <96.8 °F, heart rate >90 beats/min, respiratory rate >20 breaths/min, or white blood cell count >12 000 cells/μL or <4000 cells/μL [to convert to  × 10^9^/L, multiply by 0.001]), and at least 1 organ dysfunction sign. Organ dysfunction signs included systolic blood pressure less than 90 mm Hg, requiring mechanical ventilation, creatinine level greater than 2.0 mg/dL (to convert to micromoles per liter, multiply by 88.4) (excluding patients with *International Classification of Diseases, Ninth Revision* [*ICD-9*] or *International Statistical Classification of Diseases and Related Health Problems, Tenth Revision* [*ICD-10*] codes for end-stage renal disease [585.6 or N18.6]), total bilirubin greater than 2.0 mg/dL (to convert to micromoles per liter, multiply by 17.104), platelet count less than 100 × 10^3^ cells/μL (to convert to  × 10^9^/L, multiply by 1), or international normalized ratio greater than 1.5. Notably, our suspected sepsis definition focused on patients in whom sepsis was initially suspected but not necessarily later confirmed; this reflects the reality that distinguishing sepsis from noninfectious conditions in real-time is often challenging, yet clinicians are nonetheless expected to administer bundle-adherent care.^27^ To ensure adequate data quality, encounters without reported vital signs or missing key laboratory test results (creatinine, platelet count, and white blood cell count) within 24 hours of hospital arrival were excluded. We also excluded patients who were transferred to another acute care hospital or who were missing discharge dispositions.

SEP-1 includes lactate levels greater than 2.0 mmol/L as an additional organ dysfunction criterion, but we did not include this in our denominator owing to the known increase in lactate testing rates over time and consequent risk of bias when assessing sepsis trends.^25,28^ We also did not use administrative codes to identify sepsis because of wide variation in clinicians’ thresholds to diagnose sepsis and ongoing changes in diagnosis and coding practices over time, especially as SEP-1 was implemented when *ICD-9* transitioned to *ICD-10*.^21,29^

### Outcomes

Our primary outcome was the association of SEP-1 implementation with the combined quarterly rate of in-hospital death or discharge to hospice among patients with suspected sepsis present on admission. We label this binary outcome *short-term mortality*. We included discharge to hospice since this is an increasingly common end-of-life destination for patients with sepsis; therefore, focusing only on in-hospital mortality could produce misleading estimates of improving outcomes.^30^ Secondary outcomes included key processes of sepsis care, including lactate measurements within 24 hours of hospital arrival and administration of an anti–methicillin-resistant *Staphylococcus aureus* (MRSA) antibiotic (vancomycin, linezolid, daptomycin, telavancin, or ceftaroline) or an antipseudomonal β-lactam antibiotic (cefepime, ceftazidime, piperacillin-tazobactam, imipenem/cilastin, meropenem, doripenem, aztreonam, ceftazidime-avibactam, or ceftolozane-tazobactam) within 24 hours of hospital arrival. We focused on antibiotics and lactate measurements within 24 hours, as opposed to the 3 hours mandated by SEP-1, to provide a broader overview into changes in clinical practice and because the precise time zero defined by SEP-1 (ie, the first moment that documentation of suspected or confirmed infection, systemic inflammatory response syndrome criteria, and acute organ dysfunction all occur within a 6-hour window) is subject to high rates of interrater variability and thus difficult to capture reliably.^31^ We did not examine fluid administrations because this was not reliably captured in the data set.

### Statistical Analysis

We fit logistic regression models to assess for a change in either level (ie, an immediate post-policy change) or quarterly trends (ie, slope) for each outcome after SEP-1 implementation at a population level. Inferences were based on generalized estimating equations with robust sandwich variance estimators to account for between-hospital heterogeneity (ie, the clustering effect).^32^ The fourth quarter (Q4) of 2015 was considered a 3-month SEP-1 implementation roll-in period; as such, the pre–SEP-1 period spanned Q4 of 2013 through Q3 of 2015, and the post–SEP-1 period spanned Q1 of 2016 through Q4 of 2017. All models included time (in Qs), an indicator of the post–SEP-1 period (starting Q1 of 2016), and a 2-way interaction term between time and the post–SEP-1 period indicator to assess whether SEP-1 implementation resulted in a change in trend. When data suggested no change in trend, we also fit the model without this interaction term. For all analyses, we used logistic regression models to adjust for patients’ severity of illness and baseline characteristics, including age, sex, initial vital signs (systolic blood pressure, temperature, respiratory rate, and heart rate), and initial laboratory results (creatinine level, platelet count, bilirubin level, and white blood cell count) within 24 hours. Race, as reported by patients and recorded in each hospital’s EHR database, was also included, given its known association with sepsis outcomes, and was categorized as White, Black, or other (eg, patients who identified as Asian, Hispanic, Native American, or Pacific Islander, with multiple races, or with missing or unknown race data).^33^ We did not adjust for comorbidity burden to avoid bias from changing coding practices over time.

We conducted several sensitivity analyses. First, because not all hospitals reported data to Cerner HealthFacts during every quarter of the study period, we repeated the analyses focusing on the subset of hospitals that did report data in each quarter. Second, to assess whether SEP-1 implementation was associated with better care preventing progression to organ dysfunction and death for patients presenting with milder illness, we repeated our analyses in a broader cohort of patients with suspected serious infection (defined by ≥1 blood culture draw and intravenous antibiotic(s) within 24 hours of hospital arrival, without any requirements for SIRS or organ dysfunction). Third, we repeated our analyses in patients with suspected septic shock (defined as ≥2 SIRS criteria, ≥1 blood culture draw, and either hypotension [systolic blood pressure ≤90 mm Hg] or lactate ≥4.00 mmol/L within 24 hours of hospital arrival) because some analyses suggest that the benefit of timely sepsis bundles may be limited to this severely ill subset.^8,13,34,35,36^ Fourth, we repeated our analysis using a longer 1-year policy roll-in period (6 months prior through 6 months after October 2015, rather than just 1 Q) to account for the possibility that some hospitals may have begun preparing for SEP-1 implementation ahead of time while some may have taken longer to react.

We considered *P* < .05 to be statistically significant and used 2-tailed tests. All analyses were conducted in SAS version 9.4 (SAS Institute) and R version 3.3.1 (R Project for Statistical Computing). Data analysis was conducted between September 2020 and September 2021.

## Results

### Study Cohort and Characteristics of Patients With Suspected Sepsis on Admission

**Figure 1.**
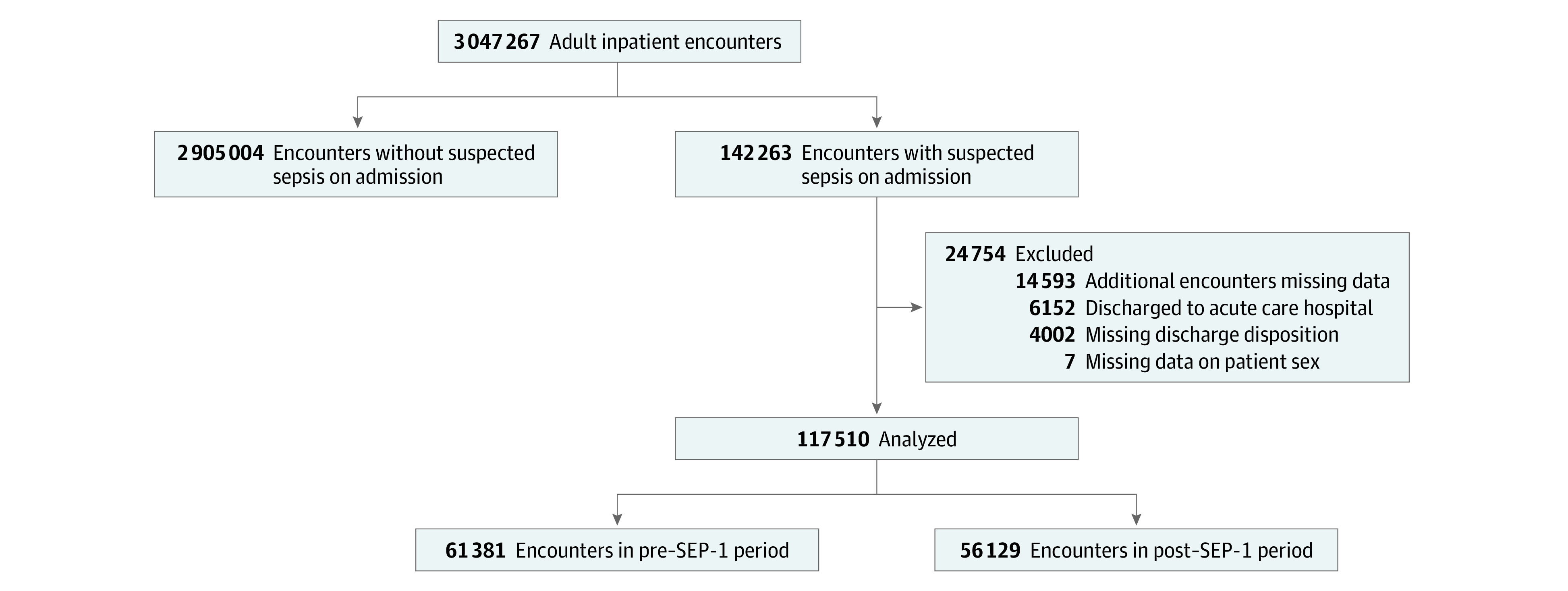
Study Cohort Flowchart Data were obtained from the Cerner HealthFacts database, which captures deidentified electronic health record patient data from geographically diverse US hospitals that use the Cerner EHR system. The final study cohort included data from 114 hospitals.

The primary study cohort included 117 510 patients (median [IQR] age, 67 years [55-78] years; 60 530 [51.5%] men and 56 980 [48.5%] women) with suspected sepsis on admission from 114 hospitals (Figure 1). The hospitals were diverse in location (58 hospitals [50.9%] in the South; 21 hospitals [18.4%] in the Northeast; 17 hospitals [14.9%] in the Midwest; 19 hospitals [16.7%] in the West), hospital size (70 hospitals [61.4%] with <200 beds; 32 hospitals [28.1%] with 200-499 beds; 12 hospitals [10.5%] with ≥500 beds), and teaching status (41 teaching hospitals [36.0%], 73 nonteaching hospitals [64.0%]). Patient characteristics and severity of illness in the pre– vs post–SEP-1 periods were generally similar (Table). Overall, comorbidities were common (median [IQR] Elixhauser comorbidity score, 13, [5-22]). Median (IQR) Sequential Organ Failure Assessment score at presentation was 4 (2-7), and 42 204 patients (35.9%) required intensive care unit care during hospitalization.

**Table.** Characteristics and Outcomes of Patients With Suspected Sepsis on Admission

Characteristic	No. (%)		
	Overall (N = 117 510)	Pre–SEP-1 (n = 61 381)^a^	Post–SEP-1 (n = 56 129)^a^
Age, median (IQR), y	67 (55-78)	67 (55-79)	66 (54-77)
Sex			
Women	56 980 (48.5)	29 981 (48.8)	26 999 (48.1)
Men	60 530 (51.5)	31 400 (51.2)	29 130 (51.9)
Race^b^			
Black	18 397 (15.7)	8510 (13.3)	9887 (17.6)
White	90 455 (77.0)	48 158 (78.5)	42 297 (73.4)
Other	8658 (7.4)	4713 (7.7)	3945 (7.0)
Comorbidities^c^			
Cancer	12 646 (10.8)	7339 (12.0)	5307 (9.5)
Congestive heart failure	33 275 (28.3)	17 302 (28.2)	15 973 (28.5)
Chronic lung disease	37 602 (32.0)	21 077 (34.3)	16 830 (30.0)
Diabetes	38 457 (32.7)	21 077 (34.3)	17 380 (31.0)
Neurologic disease	25 275 (21.5)	13 186 (21.5)	12 089 (21.5)
Kidney disease	30 910 (26.3)	15 931 (26.0)	14 979 (26.7)
Elixhauser Mortality score, median (IQR)^d^	13 (5-22)	13 (5-22)	13 (5-21)
Severity of illness, median (IQR)^e^			
SOFA score, maximum	4 (2-7)	4 (2-7)	4 (2-7)
Systolic blood pressure, mm Hg, minimum	85 (72-98)	85 (74-98)	85 (74-98)
Lactate, mmol/L, maximum	2.1 (1.3-3.6)	2.0 (1.3-3.5)	2.1 (1.4-3.7)
Creatinine, mg/dL, maximum	1.4 (0.9-2.4)	1.4 (0.9-2.4)	1.4 (0.9-2.4)
Bilirubin, mg/dL, maximum	0.7 (0.4-1.3)	0.7 (0.4-1.3)	0.7 (0.4-1.3)
Platelets, × 10^3^ cells/μL, minimum	173 (112-240)	174 (114-240)	173 (110-240)
Infectious diagnosis			
Pulmonary	53 765 (45.8)	27 989 (45.6)	25 776 (45.9)
Intraabdominal	12 309 (10.5)	6460 (10.5)	5849 (10.4)
Urinary	29 248 (24.9)	14 781 (24.1)	14 467 (25.8)
Skin or soft tissue	7336 (6.2)	5398 (8.8)	1938 (3.5)
Positive blood culture results^f^	20 806 (17.7)	10 537 (17.2)	10 269 (18.3)
Required ICU admission	42 204 (35.9)	22 147 (36.1)	20 055 (35.7)
Hospital LOS, median (IQR), d	6 (4-10)	6 (4-10)	6 (4-10)
Discharge disposition			
Home or against medical advice	65 220 (55,5)	33 595 (54.7)	31 625 (56.3)
Facility	28 380 (24.2)	15 325 (25.0)	13 055 (23.3)
Hospice	7936 (6.8)	3872 (6.3)	4064 (7.2)
In-hospital death	15 974 (13.6)	8589 (14.0)	7385 (13.2)

Abbreviations: ICU, intensive care unit; LOS, length of stay; SEP-1, Severe Sepsis and Septic Shock Early Management Bundle; SOFA, Sequential Organ Failure Assessment.

SI conversion factors: To convert bilirubin to micromoles per liter, multiply by 17.104; creatinine to micromoles per liter, multiply by 88.4; lactate to milligrams per deciliter, divide by 0.111; platelets to ×10^9^/L, multiply by 1.

^a^ The pre–SEP-1 period included the fourth quarter of 2013 through the third quarter of 2015, while the post–SEP-1 period included the first quarter of 2016 through the fourth quarter of 2017.

^b^ Race was missing or unknown in 1280 patients; these cases were assigned to the other category. The other category also included patients identified as Asian, Hispanic, Native American, Pacific Islander, or with multiple races.

^c^ Comorbidities were defined using the Elixhauser method. Cancer includes lymphoma and solid tumor with and without metastases. Diabetes includes diabetes with and without complications.

^d^ Elixhauser comorbidity scores were implemented using the Agency for Healthcare Research and Quality method.

^e^ Severity of illness variables include the worst values within 24 hours of hospital presentation. As a result of the prespecified exclusion criteria, no patients had missing vital signs, creatinine, platelet counts, or white blood cell counts within 24 hours; lactate measurements were missing in 34 292 patients (29.2%) and bilirubin was missing in 10 662 patients (9.1%).

^f^ Positive blood cultures excludes common skin contaminants.

### Changes in Lactate Measurements and Broad-spectrum Antibiotic Use

**Figure 2.**
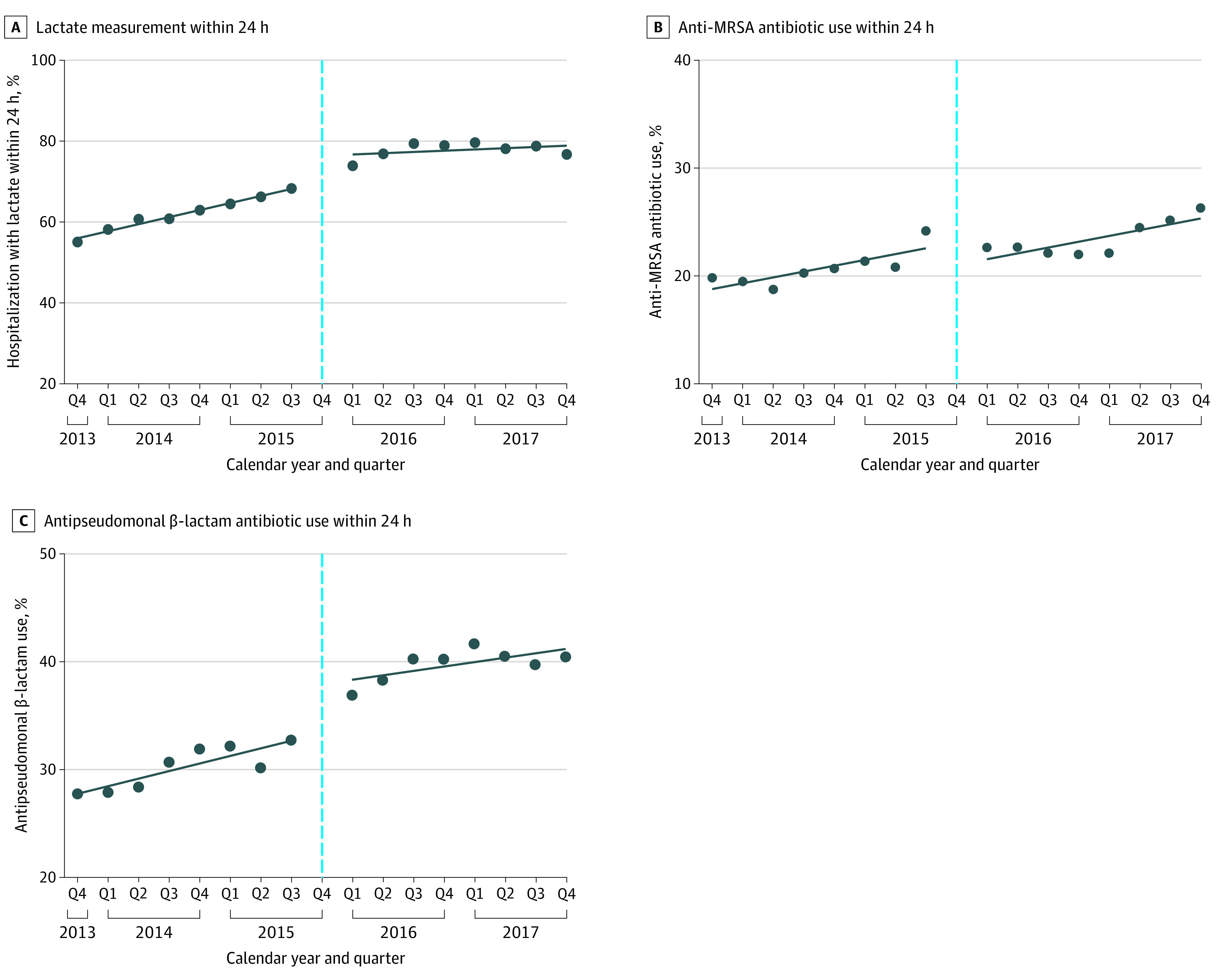
Changes in Processes of Care for Patients With Suspected Sepsis Before and After Severe Sepsis and Septic Shock Early Management Bundle (SEP-1) Implementation The vertical dotted lines indicate the SEP-1 policy implementation period in the fourth quarter (Q4) of 2015. All models included time (in Qs), an indicator of the post–SEP-1 implementation period (starting Q1 of 2016, to allow for evaluation of an immediate policy outcome), and a 2-way interaction term to assess whether SEP-1 implementation was associated with a change in trend. When data suggested no change in trend, models were also fit without this interaction term. All analyses were adjusted for patient severity of illness and baseline characteristics, including age, sex, race, initial vital signs (systolic blood pressure, temperature, respiratory rate, and heart rate), and initial laboratory results (creatinine, platelet count, bilirubin, and white blood cell count) if assessed within 24 hours.

Unadjusted rates of lactate testing within 24 hours for patients with suspected sepsis increased from 55.1% (95% CI, 53.9%-56.2%) in Q4 of 2013 to 76.7% (95% CI, 75.4%-78.0%) in Q4 of 2017, and overall 61.9% (95% CI, 61.5%-62.3%) in the pre–SEP-1 period vs 77.9% (95% CI, 77.6%-78.3%) in the post–SEP-1 period (crude odds ratio [OR], 2.17; 95% CI, 2.12-2.23). SEP-1 implementation was not associated with changes in quarterly risk-adjusted lactate testing trends (adjusted OR [aOR], 0.95; 95% CI, 0.88-1.03) (Figure 2A). There was no statistically significant immediate level change in risk-adjusted lactate testing rates after SEP-1 implementation (aOR, 2.17; 95% CI, 0.88-5.36); however, after removing the interaction term for potential trend change, the immediate level change was statistically significant (aOR, 1.34; 95% CI, 1.04-1.74).

Unadjusted rates of anti-MRSA antibiotic administration within 24 hours increased from 19.8% (95% CI, 18.9%-20.7%) in Q4 of 2013 to 26.3% (95% CI, 24.9%-27.7%) in Q4 of 2017 and 20.6% (95% CI, 20.3%-20.9%) overall in the pre–SEP-1 period vs 23.2% (95% CI, 22.8%-23.5%) in the post–SEP-1 period (crude OR, 1.16; 95% CI, 1.13-1.20). Similarly, unadjusted rates of antipseudomonal antibiotic administration increased from 27.7% (95% CI, 26.7%-28.8%) in Q4 of 2013 to 40.4% (95% CI, 38.9%-42.0%) in Q4 of 2017 and 30.1% (95% CI, 29.8%-30.5%) overall in the pre–SEP-1 period vs 39.8% (95% CI, 39.4%-40.2%) in the post–SEP-1 period (crude OR, 1.53; 95% CI, 1.50-1.57). However, SEP-1 implementation was not associated with an immediate level change in risk-adjusted anti-MRSA antibiotic administration rates (aOR, 0.93; 95% CI, 0.47-1.85) or antipseudomonal antibiotic administration rates (aOR, 1.38; 95% CI, 0.83-2.31) nor a change in quarterly risk-adjusted administration rates (anti-MRSA antibiotics: aOR, 1.00; 95% CI, 0.93-1.07; antipseudomonal antibiotics: aOR, 0.99; 95% CI, 0.94-1.04) (Figure 2B and C). After removing the interaction term for potential trend change, there was still no significant immediate level change for either outcome.

### Changes in Mortality

**Figure 3.**
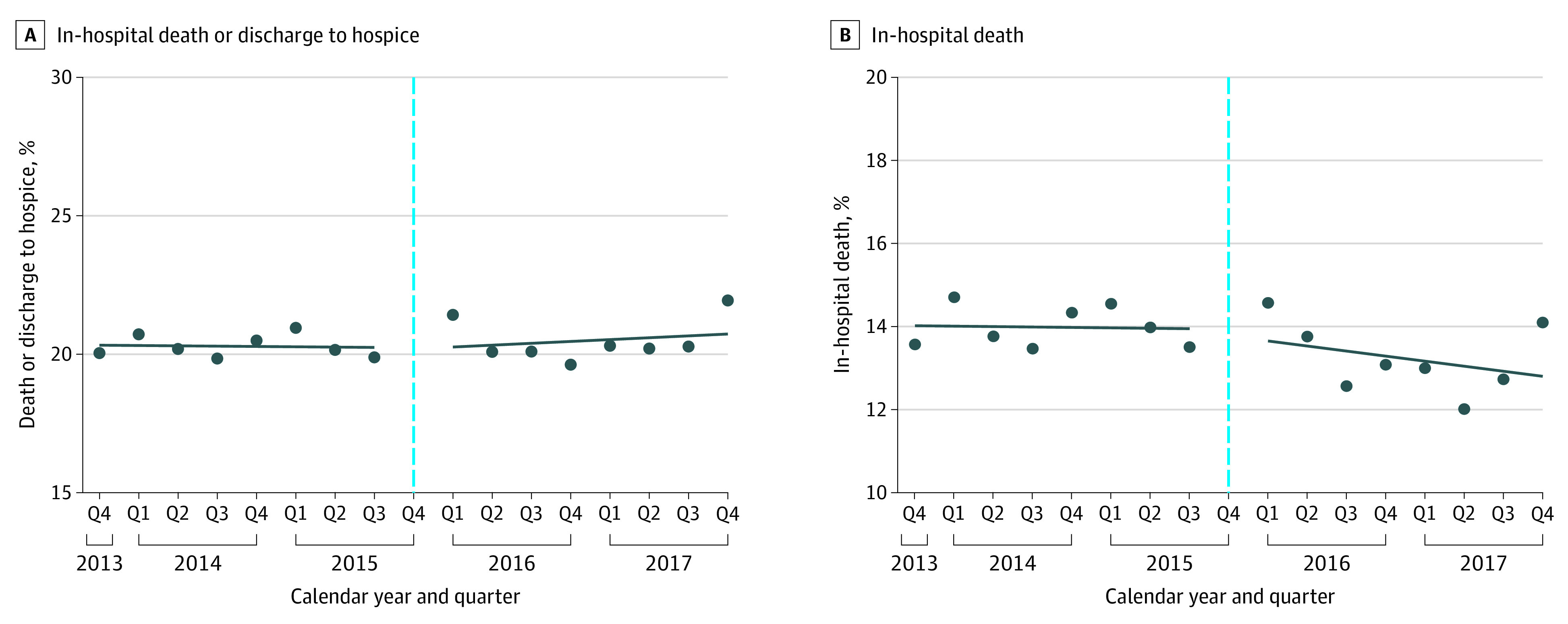
Changes in Risk-Adjusted Outcomes of Patients With Suspected Sepsis Before and After Severe Sepsis and Septic Shock Early Management Bundle (SEP-1) Implementation The vertical dotted lines denote the SEP-1 policy implementation period in the fourth quarter (Q4) of 2015. Models included time (in Qs), an indicator of the post–SEP-1 implementation period (starting in Q1 of 2016, to allow for evaluation of an immediate policy outcome), and a 2-way interaction term to assess whether SEP-1 implementation was associated with a change in trend. Analyses were adjusted for patient severity of illness and baseline characteristics, including age, sex, race, initial vital signs (systolic blood pressure, temperature, respiratory rate, and heart rate), and initial laboratory results (creatinine, platelet count, bilirubin, and white blood cell count) if assessed within 24 hours.

The unadjusted rate of short-term mortality for patients with suspected sepsis did not change during the study period: 20.0% (95% CI, 19.2%-21.0%) in Q4 of 2013 and 21.9% (95% CI, 20.7%-23.3%) in Q4 of 2017, and overall 20.3% (95% CI, 20.0%-20.6%) in the pre–SEP-1 period vs 20.4% (95% CI. 20.1%-20.7%) in the post–SEP-1 period (crude OR, 1.01; 95% CI, 0.98-1.04). There was no association between SEP-1 implementation and risk-adjusted short-term mortality rates for either an immediate level change (aOR, 0.94; 95% CI, 0.70-1.29]) or quarterly trends (aOR, 1.00; 95% CI, 0.97-1.04) (Figure 3). After removing the interaction term representing a potential trend change, there was still no significant level change.

When considering in-hospital mortality and discharge to hospice separately, unadjusted in-hospital mortality decreased from 14.0% (95% CI, 13.7%-14.3%) in the pre–SEP-1 period to 13.2% (95% CI, 12.9%-13.4%) in the post–SEP-1 period (crude OR, 0.93; 95% CI, 0.91-0.96), whereas discharge to hospice rates increased from 6.3% (95% CI, 6.1%-6.5%) to 7.2% (95% CI, 7.0%-7.5%) (crude OR, 1.16; 95% CI, 1.11-1.21).

### Sensitivity Analyses

Among 26 hospitals that reported data in each study quarter (45 871 patients with suspected sepsis), findings were similar to the main analysis: SEP-1 implementation was associated with a significant immediate level increase in risk-adjusted lactate testing rates (aOR, 1.62; 95% CI, 1.03-2.54), but no significant change in quarterly trends in lactate testing nor in level or trends for broad-spectrum antibiotic use or short-term mortality (eFigure 1 in the Supplement). Results were also similar in the full hospital cohort when expanding the analyses to a broader population of patients with suspected serious infections (based on blood culture orders and intravenous antibiotics), when limiting the analysis to patients with suspected sepsis shock, and when considering a longer 1-year policy roll-in period (eFigures 2-4 in the Supplement).

## Discussion

This cohort study evaluated sepsis treatment patterns and outcomes among 117 510 patients admitted to 114 US hospitals with clinical evidence of suspected sepsis from 2 years before through 2 years after implementation of the CMS SEP-1 measure. We found that SEP-1 implementation in Q4 of 2015 was associated with an immediate increase in lactate testing but no change in a preexisting trend toward increasing use of broad-spectrum antibiotics and no improvements in the combined outcome of in-hospital death or discharge to hospice. These findings were consistent in multiple sensitivity analyses, including patients with septic shock alone, and an analysis incorporating a 1-year policy roll-in period.

Our findings are concordant with a recent analysis that also found no association between SEP-1 implementation and sepsis-associated mortality in 11 hospitals in the University of Pittsburgh Medical Center health care system despite substantial increases in lactate testing and modest increases in fluid and broad-spectrum antibiotic administration.^37^ Our study expands on that analysis using a larger and more diverse set of hospitals, including many small, nonteaching hospitals. The congruence of these 2 studies provides greater confidence in the conclusion that SEP-1 implementation has not led to improvements in sepsis mortality. We further did not detect a change in mortality among a broader cohort of patients with suspected serious infections (ie, those with blood culture orders and intravenous antibiotics but without requiring SIRS or organ dysfunction), suggesting that SEP-1 also did not reduce progression of sepsis and death in patients with less severe illness at presentation.

Our findings differ from several other studies that reported substantial reductions in sepsis mortality after bundle implementations.^15,16,17,18^ The most relevant comparison to SEP-1 may be the mandated sepsis protocols in New York state (known as *Rory’s Regulations*); several analyses have associated these with significant improvements in mortality over time and compared with similar states without sepsis regulations.^19,20^ However, SEP-1 and Rory’s Regulations may not be directly comparable, as Rory’s Regulations included a more comprehensive set of interventions, including standardized protocols at each hospital, aggressive awareness and education campaigns, and mandates for public reporting of both bundle adherence and outcomes. This has important implications for sepsis quality improvement efforts around the world, as it suggests that intensive efforts beyond simply requiring public reporting of bundle adherence rates may be necessary to substantially improve outcomes. However, the implementation of the New York state initiative was also associated with a substantial increase in the number of reported sepsis cases, suggesting that some of the decrease in mortality may have been due to more ascertainment of patients who were less severely ill.^38,39,40^ We designed our study to minimize ascertainment bias by using objective clinical criteria for sepsis rather than clinician diagnoses or administrative codes.

The focus on in-hospital mortality alone in prior sepsis bundle time-series analyses may be another source of bias, as more and more patients, including patients admitted with sepsis, are discharged to hospice for end-of-life care rather than dying in-hospital.^30,41,42^ Consistent with this national trend, we observed lower in-hospital mortality rates but higher rates of hospice discharge during the post–SEP-1 period.

We found that SEP-1 implementation was associated with an immediate increase in lactate testing. This suggests that increasing lactate testing can be quickly acted on by hospitals, compared with other care processes that may be substantially more complex to change. Indeed, prior studies have demonstrated that computerized alerts and standardized order sets can lead to immediate increases in lactate testing.^18,43^ However, lactate measurements alone are unlikely to improve outcomes. Indeed, a randomized clinical trial of lactate measurements vs bedside perfusion assessments to guide resuscitation of septic shock found no difference in mortality with the lactate strategy.^44^ We also found that broad-spectrum antibiotic use was increasing before SEP-1 implementation, despite major national and local stewardship efforts during the past decade, and implementation of SEP-1 was not associated with a change in this trend. Other studies predating SEP-1 have shown steadily increasing rates of lactate testing and broad-spectrum antibiotic use in patients with suspected sepsis.^28,45^ Both trends may reflect the growing influence of the Surviving Sepsis campaign guidelines, which have been emphasizing these processes of care for nearly 2 decades.^46,47^ However, some recent studies have documented increases in broad-spectrum antibiotic use specifically associated with SEP-1 implementation, suggesting hospitals’ responses to this policy have varied.^48,49^

Numerous observational studies have found associations between bundle adherence and lower mortality for patients with sepsis, including a recent national study of Medicare beneficiaries using SEP-1 data.^35,50,51^ However, these analyses are subject to many unmeasured potential confounders that could lead to overestimates of the bundle’s association with mortality.^6,13,52^ We emphasize that our study did not directly address whether or not adherence to the SEP-1 bundle was associated with better sepsis outcomes at an individual patient level. Rather, our analysis was designed to evaluate SEP-1 implementation as a policy by assessing changes in population-level sepsis outcomes.

### Limitations

Our study has several limitations. First, our cohort was drawn from hospitals that used a common EHR system, potentially limiting generalizability. However, a strength of our study is that hospitals were not limited to a single health care system and were diverse in geographic region, size, and teaching status. Second, we were unable to investigate trends in fluid administration owing to data set limitations. This is an important topic for additional research, as SEP-1’s requirement for 30 mL/kg of fluids within 3 hours of sepsis-associated hypotension or lactic acidosis is controversial, since this intervention has not been consistently associated with improved outcomes.^35,53,54,55^ Third, we assessed trends in broad-spectrum antibiotics and lactate testing within 24 hours, rather than within 3 hours as mandated by SEP-1. We did this to obtain broader insight into changes in processes of care and because of the imprecision in defining and identifying time zero.^31^ Fourth, most hospitals in the data set did not report data in each study quarter. For this reason, we conducted a sensitivity analysis among consistently reporting hospitals and found similar results as the main analysis. Fifth, the data set did not allow us to assess any posthospitalization outcomes. Sixth, it is possible that some hospitals may not have fully reacted to SEP-1 until adherence rates became publicly available on the CMS Hospital Compare website in July 2018, but we only had data available through the end of 2017. Investigating whether or not the shift to report SEP-1 data on hospital care was associated with improvements in sepsis outcomes is an important topic for future research.

## Conclusions

In this cohort study, SEP-1 implementation in October 2015 was associated with an immediate increase in lactate testing rates, no significant change in already-increasing rates of broad-spectrum antibiotic use, and no change in the combined outcome of in-hospital death or discharge to hospice for patients with clinical evidence of suspected sepsis admitted to 114 US hospitals between 2013 and 2017. These findings suggest that alternate approaches to improving mortality for patients with sepsis are warranted.

## Notes

Supplement. **eFigure 1.** Sensitivity Analysis Limited to Patients with Suspected Sepsis in Consistent-Reporter Hospitals **eFigure 2.** Sensitivity Analysis Focusing on a Broader Definition of Suspected Sepsis in Full Hospital Cohort **eFigure 3.** Sensitivity Analysis Limited to Patients With Suspected Septic Shock in Full Hospital Cohort **eFigure 4.** Sensitivity Analysis Using a 1-Year Policy Roll-In Period for Patients With Suspected Sepsis in Full Hospital Cohort
