# Supplementary material for: Association Between Implementation of the Severe Sepsis and Septic Shock Early Management Bundle Performance Measure and Outcomes in Patients With Suspected Sepsis in US Hospitals
Source: JAMA Netw Open. 2021 Dec 20;4(12):e2138596. doi: 10.1001/jamanetworkopen.2021.38596 (PMC8689388; doi:10.1001/jamanetworkopen.2021.38596)
Supplement: Supplement. — eFigure 1. Sensitivity Analysis Limited to Patients with Suspected Sepsis in Consistent-Reporter Hospitals eFigure 2. Sensitivity Analysis Focusing on a Broader Definition of Suspected Sepsis in Full Hospital Cohort eFigure 3. Sensitivity Analysis Limited to Patients With Suspected Septic Shock in Full Hospital Cohort eFigure 4. Sensitivity Analysis Using a 1-Year Policy Roll-In Period for Patients With Suspected Sepsis in Full Hospital Cohort [file jamanetwopen-e2138596-s001.pdf]

Supplemental Online Content

Rhee C, Yu T, Wang R, et al; for the CDC Prevention Epicenters Program. Association between implementation of the Severe Sepsis and Septic Shock Early Management Bundle performance measure and outcomes in patients with suspected sepsis in US hospitals. *JAMA Netw Open*. 2021;4(12):e2138596. doi:10.1001/jamanetworkopen.2021.38596

eFigure 1. Sensitivity Analysis Limited to Patients with Suspected Sepsis in Consistent-Reporter Hospitals

eFigure 2. Sensitivity Analysis Focusing on a Broader Definition of Suspected Sepsis in Full Hospital Cohort

eFigure 3. Sensitivity Analysis Limited to Patients With Suspected Septic Shock in Full Hospital Cohort

eFigure 4. Sensitivity Analysis Using a 1-Year Policy Roll-In Period for Patients With Suspected Sepsis in Full Hospital Cohort

This supplemental material has been provided by the authors to give readers additional information about their work.

eFigure 1. Sensitivity Analysis Limited to Patients with Suspected Sepsis in Consistent-Reporter Hospitals

(n=45,871 patients meeting the primary sepsis definition among 26 hospitals that reported data in each quarter of study period).

A

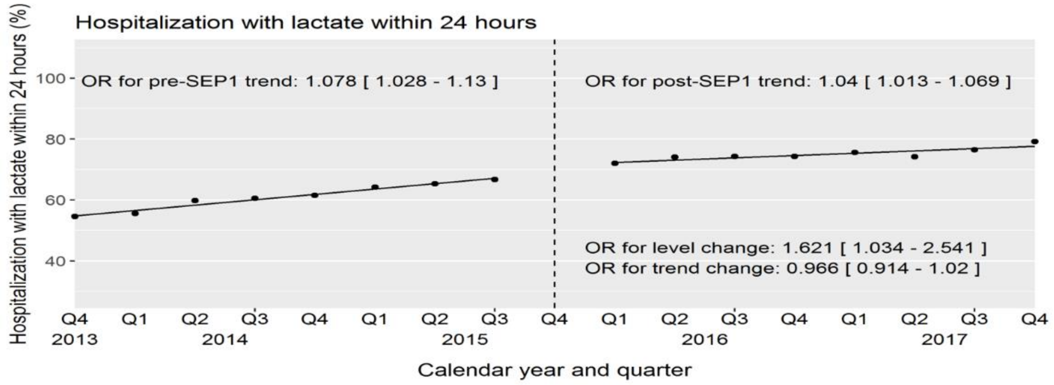

B

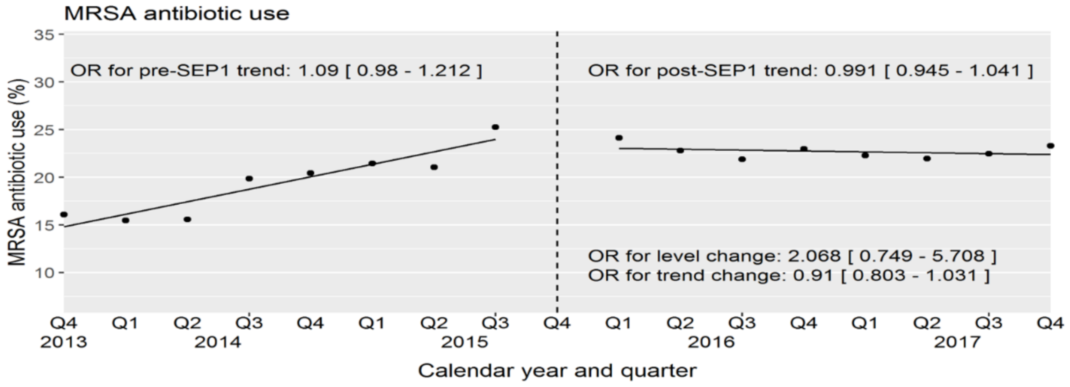

C

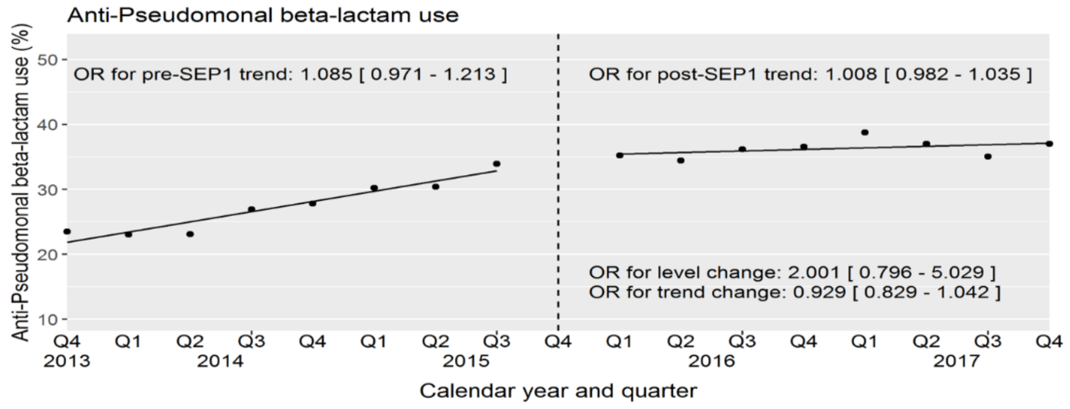

© 2021 Rhee C et al. *JAMA Network Open.*

D

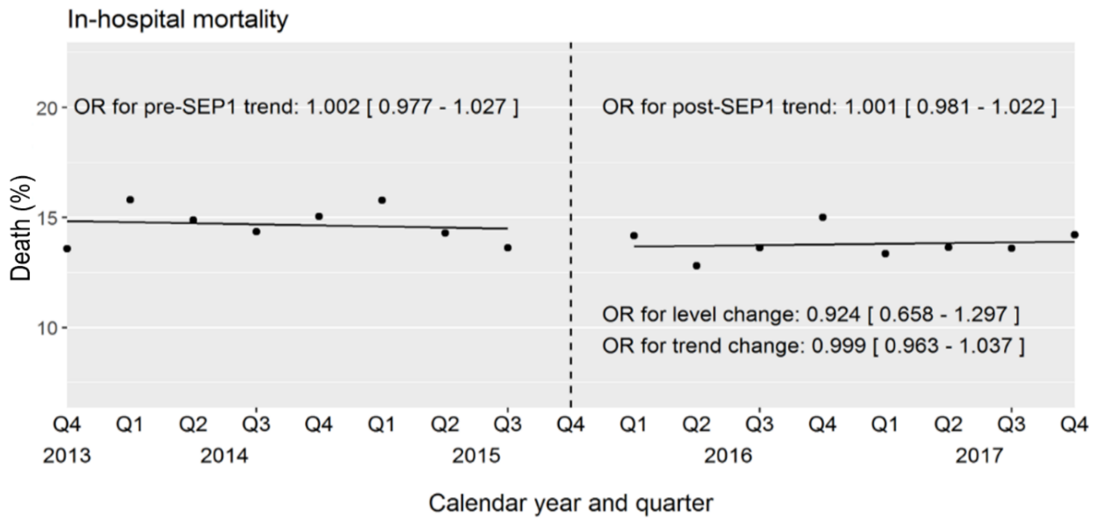

E

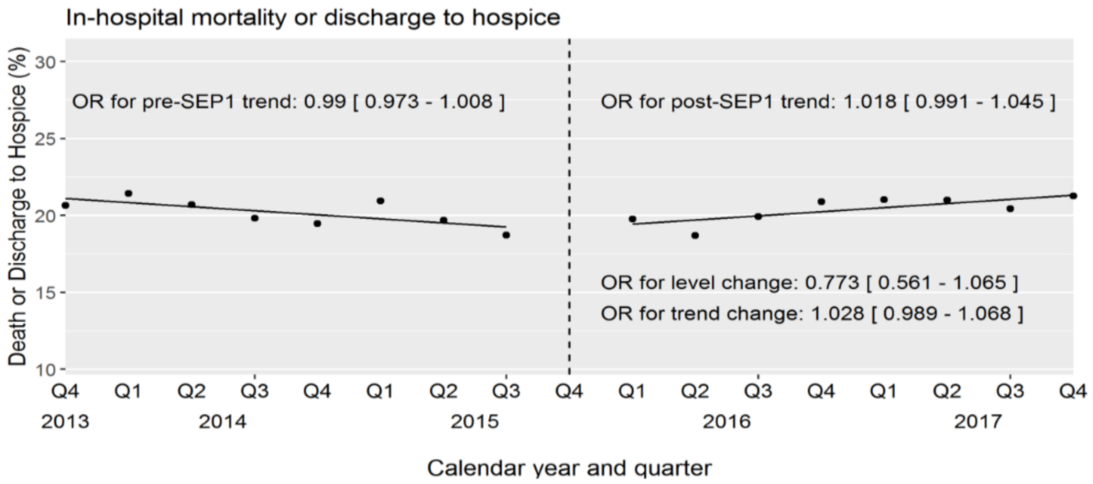

© 2021 Rhee C et al. *JAMA Network Open.*

eFigure 2. Sensitivity Analysis Focusing on a Broader Definition of Suspected Sepsis in Full Hospital Cohort

(n=289,114 patients among 118 hospitals). The definition used for suspected sepsis in this analysis was blood cultures and intravenous antibiotics within 24 hours, without requirements for systemic inflammatory response syndrome criteria or organ dysfunction.

A

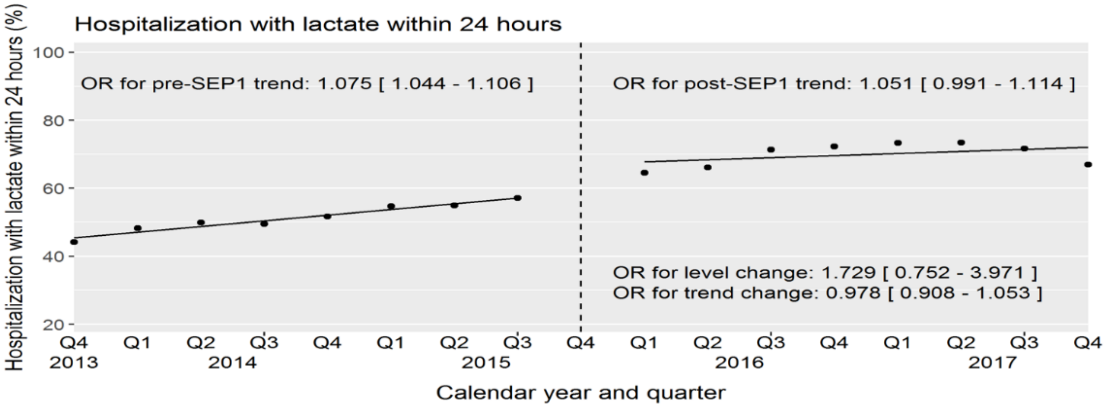

B

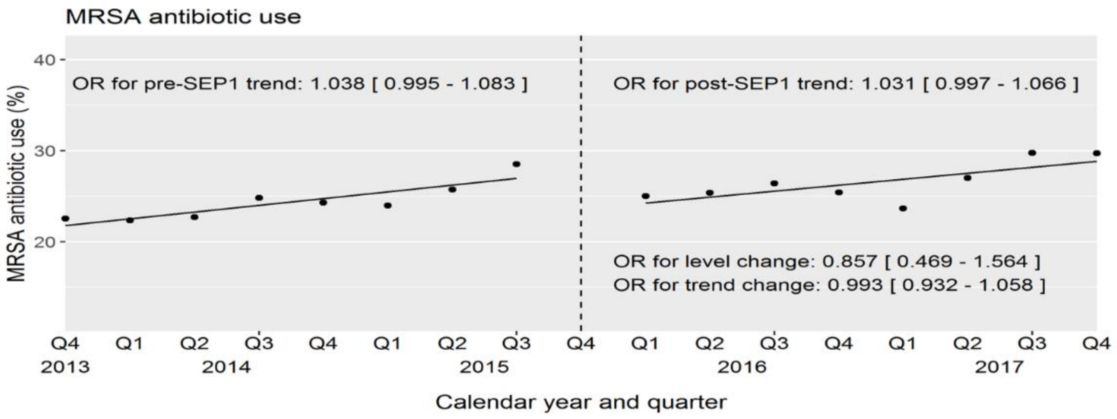

C

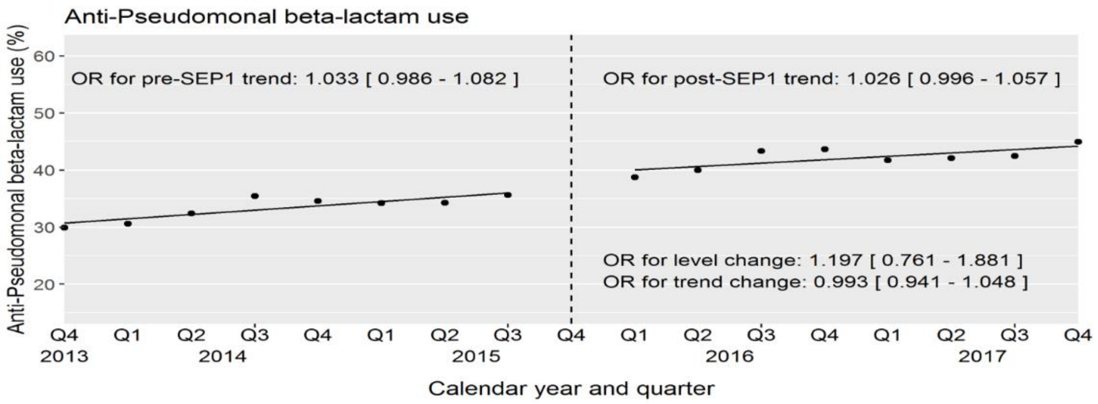

© 2021 Rhee C et al. *JAMA Network Open.*

D

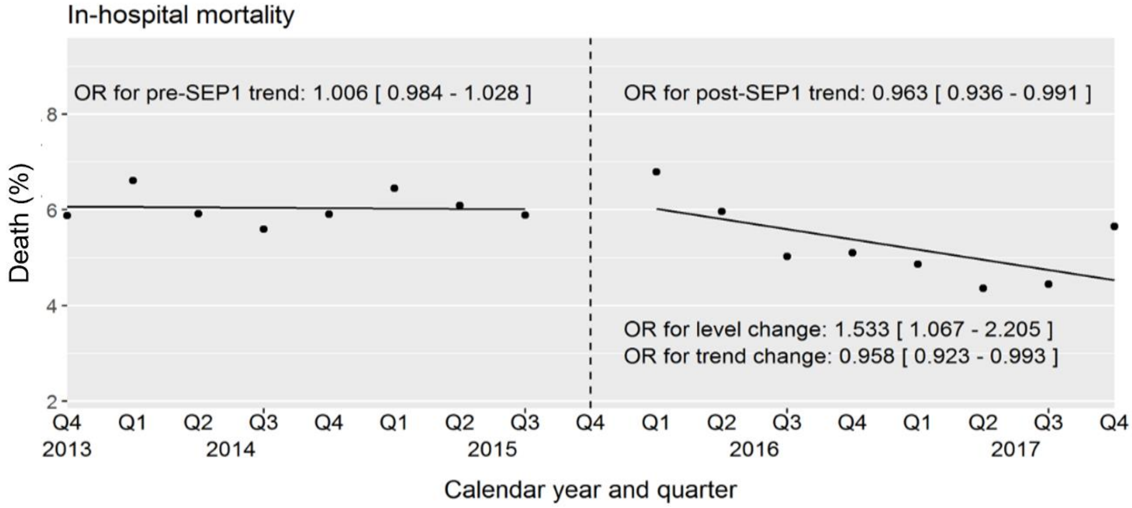

E

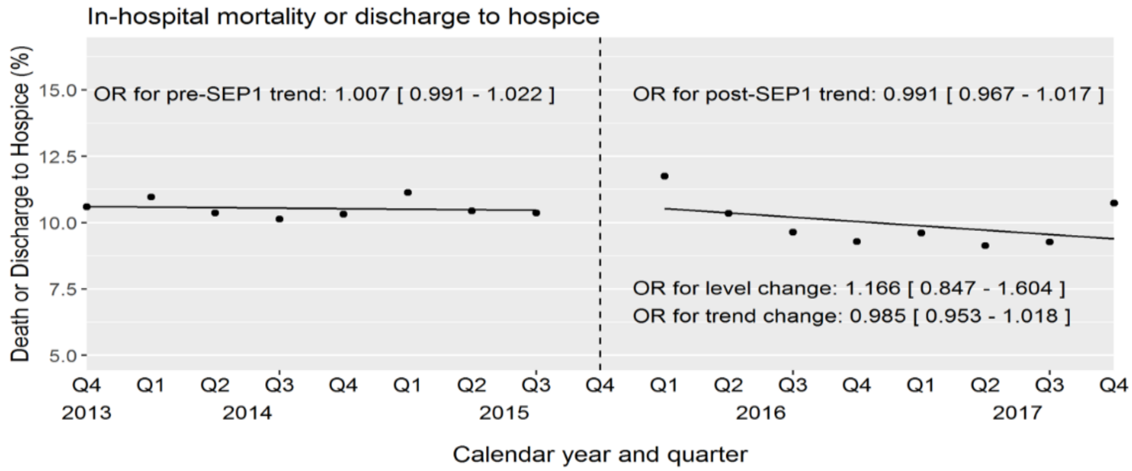

© 2021 Rhee C et al. *JAMA Network Open.*

eFigure 3. Sensitivity Analysis Limited to Patients With Suspected Septic Shock in Full Hospital Cohort

(n=27,097 patients among 111 hospitals). Suspected septic shock was defined as ≥2 SIRS criteria, a blood culture order, and either a systolic blood pressure < 90 mmHg or a lactate level ≥4.0 mmol/L within 24 hours of hospital arrival.

A

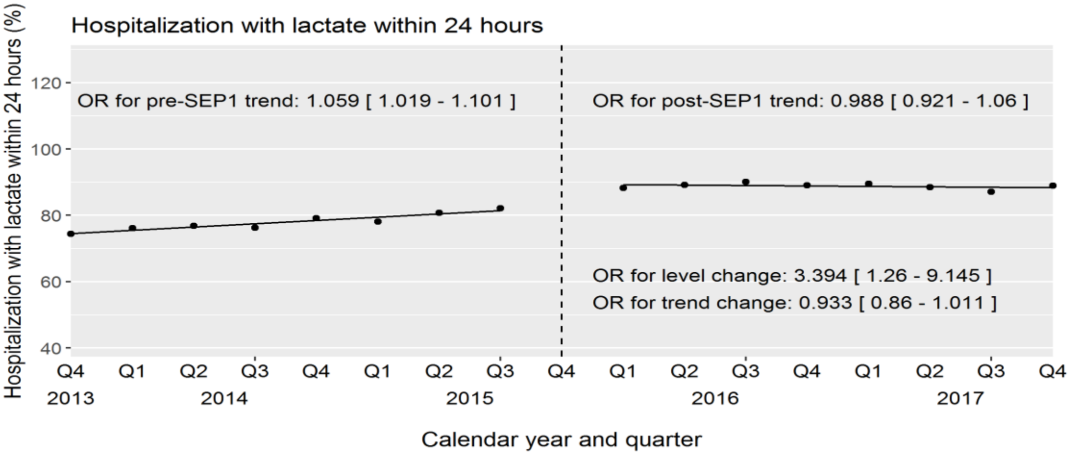

B

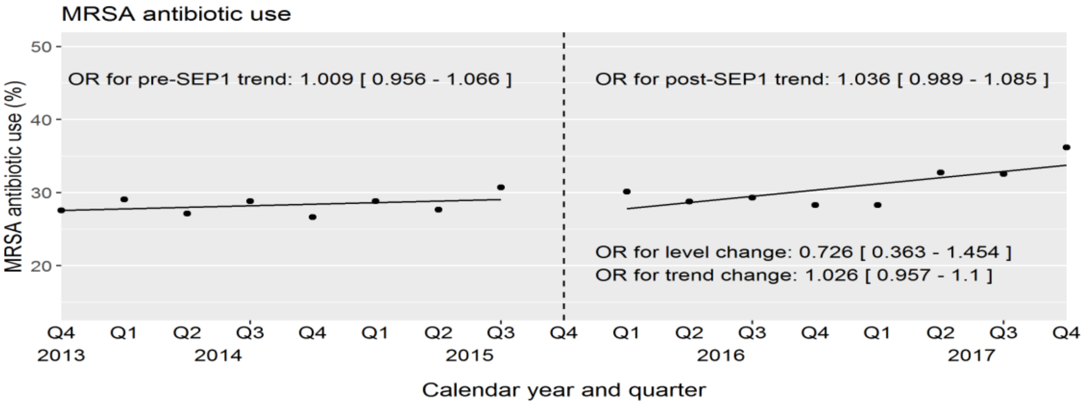

C

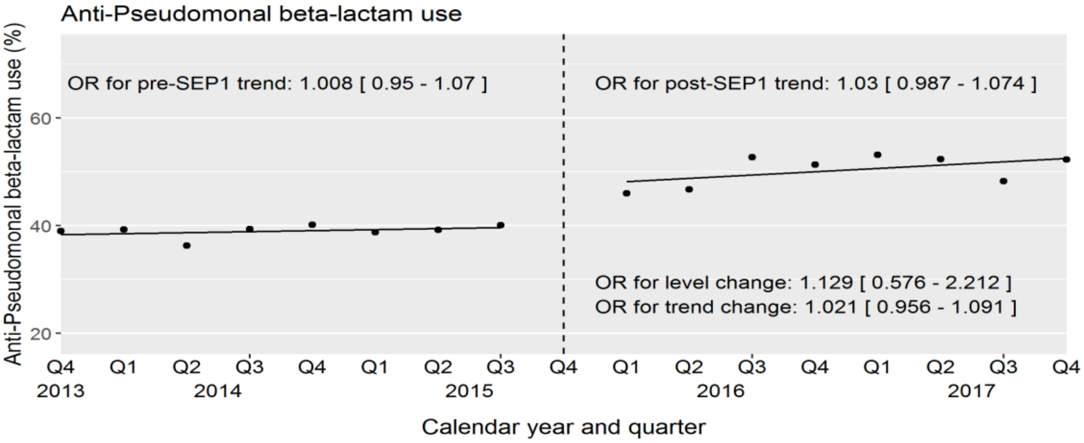

© 2021 Rhee C et al. *JAMA Network Open.*

D

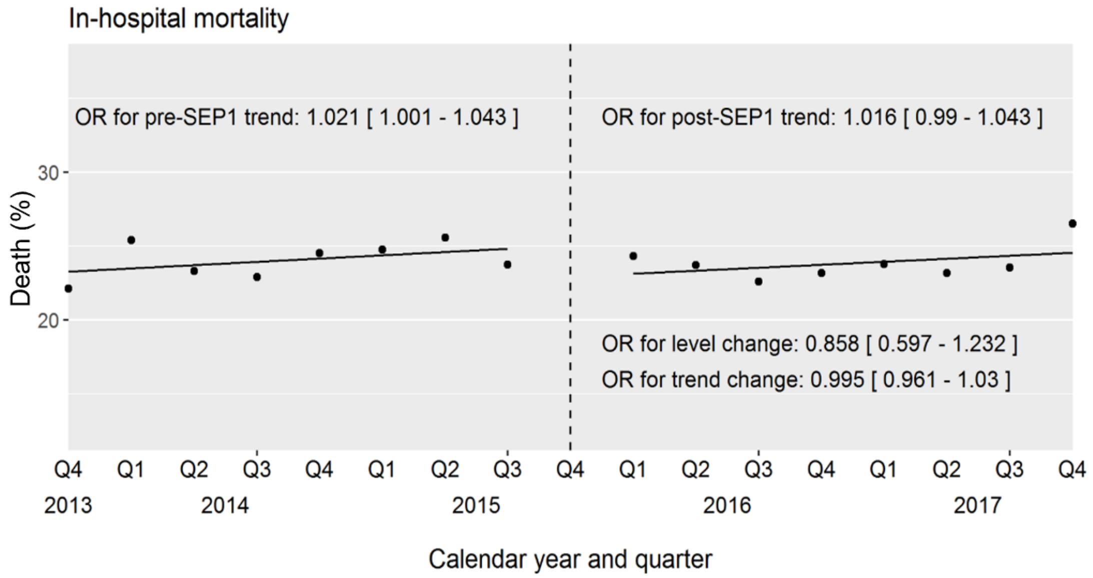

E

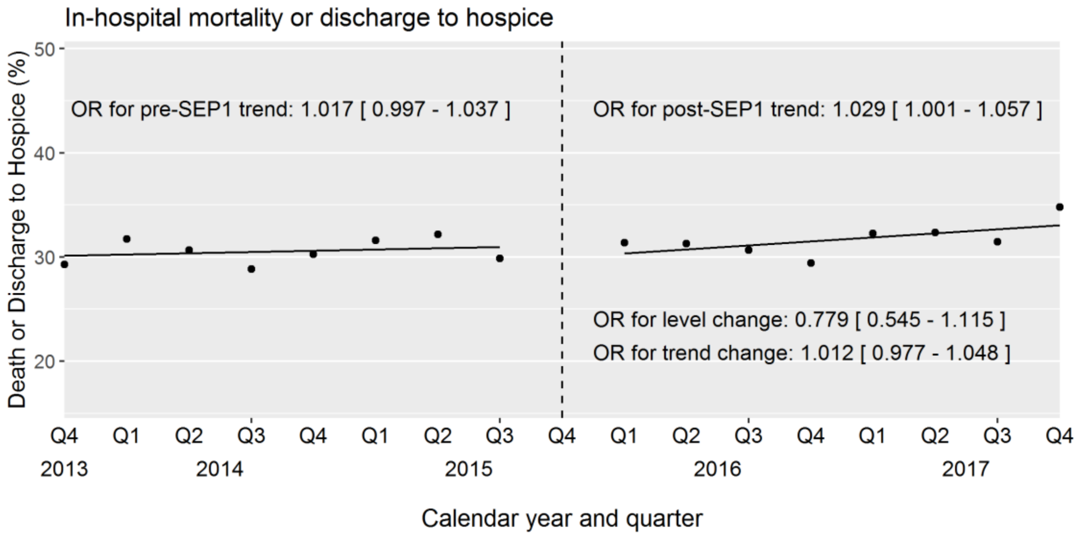

© 2021 Rhee C et al. *JAMA Network Open.*

eFigure 4. Sensitivity Analysis Using a 1-Year Policy Roll-In Period for Patients With Suspected Sepsis in Full Hospital Cohort

(n=96,244 patients among 113 hospitals). This analysis used a one-year policy roll-in period (6 months prior through 6 months after October 2015, rather than just one quarter) to account for the possibility that some hospitals may have begun preparing for SEP-1 implementation ahead of time while some may have taken longer to react.

A

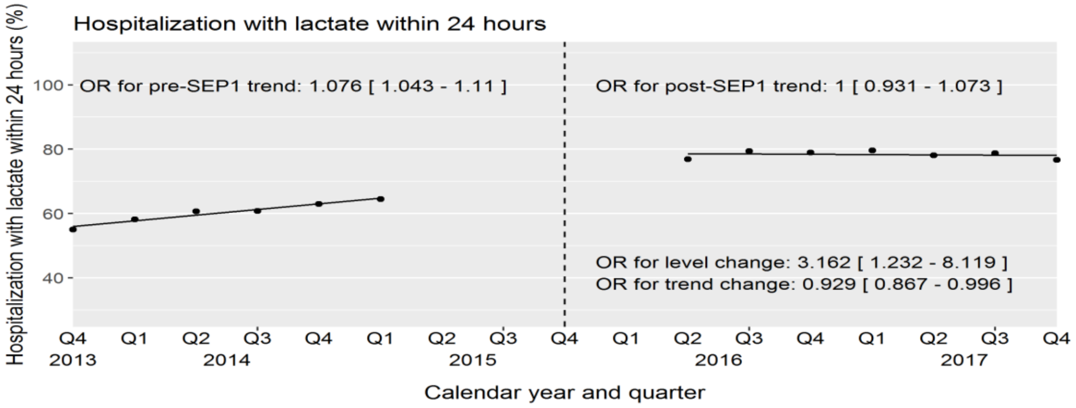

B

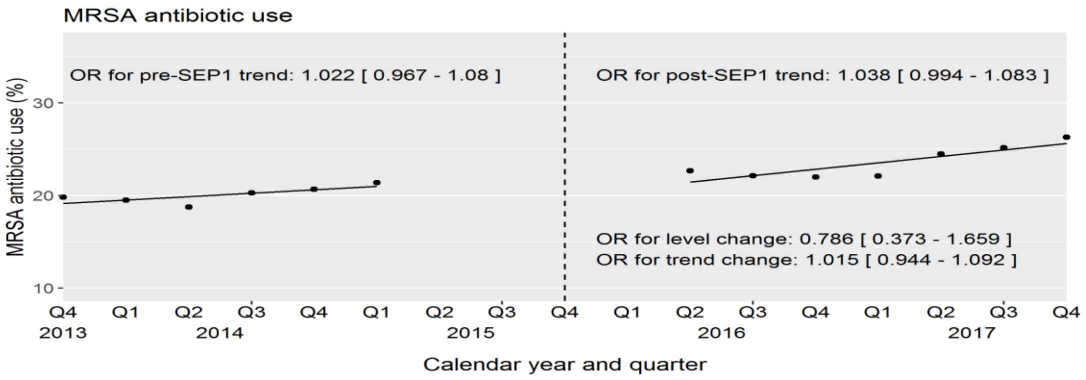

C

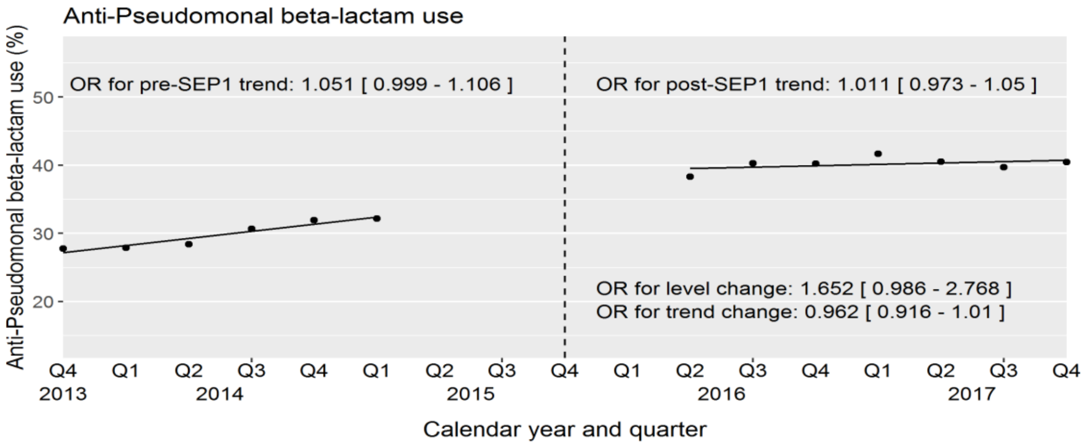

© 2021 Rhee C et al. *JAMA Network Open.*

D

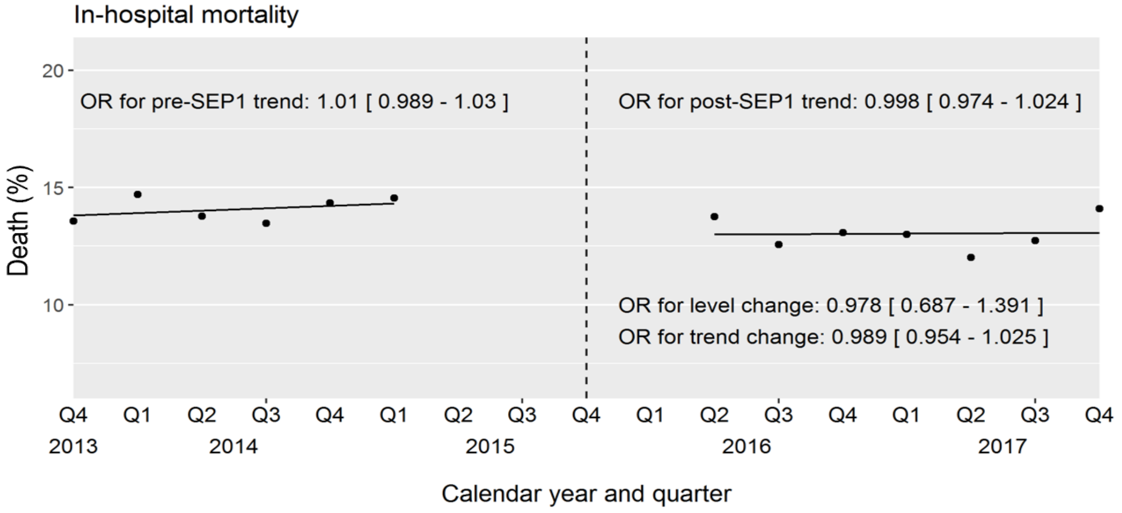

E

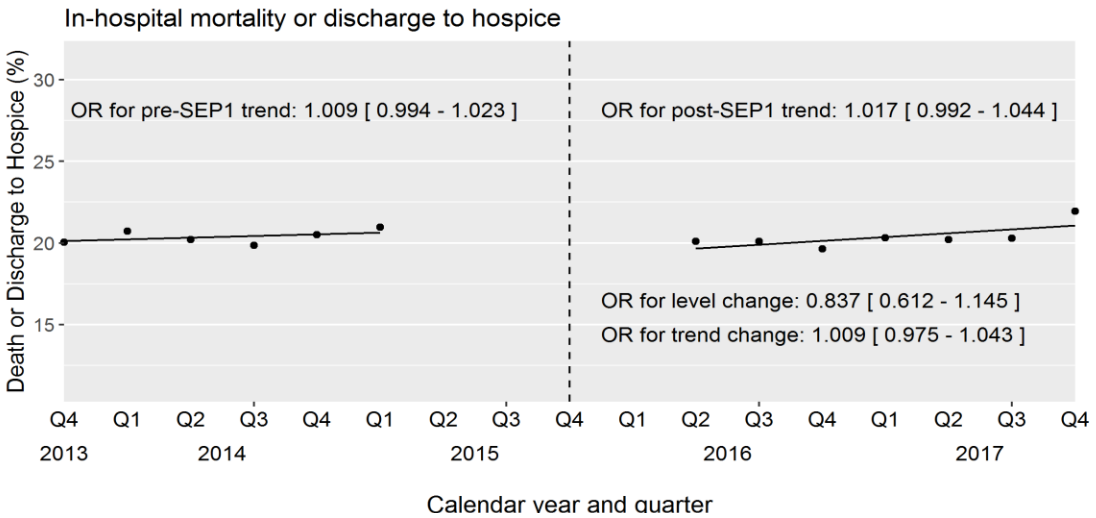

© 2021 Rhee C et al. *JAMA Network Open.*
